# Girdin acts as an oncogene in gastric cancer by regulating AKT/GSK3β/β-catenin signaling

**DOI:** 10.1007/s10142-022-00927-8

**Published:** 2023-01-06

**Authors:** Yun Wang, Qiang Fu, Yun-jian Tao, Sheng-nan Ying, Heng-gao Zhong, Yue Zhu, Xiao-han Qian, Lin Miao, Li-hua Yang

**Affiliations:** 1 Department of Digestive Medicine, Second Affiliated Hospital of Nanjing Medical University, No. 121 Jiang Jia Yuan Road, Nanjing, China; 2 Department of Digestive Medicine, Jiangsu Rudong County People’s Hospital, Nanjing, China

**Keywords:** Gastric cancer, Cell proliferation, Cell invasion, β-catenin

## Abstract

ThE present work focused on exploring Girdin expression within gastric cancer (GC), examining the effect of Girdin on the cell phenotype of GC, and clarifying the underlying mechanisms. Girdin expression in GC samples was identified by immunohistochemistry (IHC) and quantitative real-time PCR (qRT-PCR) assays. Girdin-targeting siRNAs were transfected into GC cells; later, we examined GC cell proliferation, migration, invasion, and apoptosis, respectively. Additionally, the protein expression was examined through Western blotting assay. Moreover, the tumor implantation experiment was conducted for examining Girdin knockdown in vivo. The results showed that Girdin expression elevated within GC samples, which was associated with the dismal prognostic outcome. Girdin knockdown suppressed GC cell proliferation, migration, and invasion, and enhanced apoptosis and cell cycle arrest. Girdin promoted the phosphorylation of AKT, GSK3β, and β-catenin. Moreover, Girdin inhibited the phosphorylation of β-catenin. Girdin suppressed cell apoptosis and stimulated cell migration and invasion, while AKT inhibitor (MK2206) treatment reversed the effect of Girdin overexpression, and GSK3β inhibitor (CHIR99021) treatment enhanced the effect of Girdin overexpression on GC cells. Besides, Girdin delayed tumor growth in vivo. In conclusion, Girdin was abnormally expressed in GC samples, which promoted the development of GC by regulating AKT/GSK3β/β-catenin signaling.

## Introduction

Gastric cancer (GC) ranks the 3^rd^ and 4^th^ places in terms of its mortality and morbidity in the world, which has seriously threatened human health (Rausei and Lianos 2020). The Office of Cancer Prevention and Control of China has conducted an analysis and research on GC. As shown by the statistical results, GC is the critical feature of tumor genesis, while the rapidly proliferating and metastasizing tumor cells are tightly associated with dismal prognostic outcome in cancer patients (Song et al. 2018; Strand et al. 2017). Generally, GC is mainly derived from the stromal epithelium, and this type of GC is usually called gastric adenocarcinoma. Adenocarcinoma represents a frequently occurring GC subtype, which takes up 95% of all GC cases (Duarte et al. 2018; Laks et al. 2017). Although gastroscopy can be employed to diagnose GC in the early stage, many GC cases are at the middle or advanced stage when they are diagnosed due to the rapid tumor proliferation and metastasis. As a result, these patients have disappointed prognostic outcomes and poor 5-year survival after operation. As shown in some studies, the early diagnosis of GC can significantly improve the patient survival (Wang et al. 2019; Wu et al. 2019).

Tumor biomarkers have been identified to be tightly associated with tumor genesis and progression (Hu et al. 2020). Tumor biomarkers are present in or are produced by tumors. In recent years, tumor markers have been extensively used to effectively distinguish tumor tissue from normal tissue, and to determine the existence of tumor by detecting tumor marker concentrations in the blood or body fluid of patients (So 2020). Therefore, it is of significant importance to discover new biomarkers to diagnose and treat GC in early stage. Moreover, detection of specific immunohistochemical markers can also improve the early diagnosis, treatment, and even prognosis of GC patients (Lili et al. 2019).

Galpha-interacting vesicle-associated protein (GIV, aka Girdin) is a guanine exchange factor (GEF) for the trimeric G protein Galphai and a bona fide metastasis-related gene (Dunkel et al. 2016). Our team found that Girdin was highly expressed in GC and other tumors, such as lung cancer, breast cancer (BC), and pancreatic cancer, which predicted the poor prognosis of tumors (Li et al. 2019; Biehler et al. 2020; Lu et al. 2018). Dunkel et al. have reported that Girdin can serve as an effective prognosticator in breast cancer (Dunkel et al. 2016). Several studies have shown that Girdin is also a biomarker of great prognostic significance in colon cancer (Ghosh et al. 2016; Barbazan et al. 2016). It has been reported that Girdin participates in the regulating processes of DNA damage-induced apoptosis (Chen et al. 2020), cell adhesion and cytoskeletal organization (Wang et al. 2018a), tumor cell stemness (Natsume et al. 2012), and collective invasion of cancer cells (Wang et al. 2016). Moreover, Wang et al. have reported that Girdin is over-expressed in GC tumor tissue, and is positively correlated with tumor invasion depth and lymph node metastasis (Wang et al. 2014).

Our study focused on exploring the expression level of Girdin in GC, examining the Girdin function in GC cell phenotype, and clarifying the exact mechanisms.

## Methods

### Patient and tissue samples

We acquired altogether 57 GC and 57 matched adjacent non-carcinoma tissues from GC cases undergoing surgery at our hospital (Nanjing, China) from January 2014 to December 2017 (Table 1). After collection, each tissue sample was frozen within liquid nitrogen at once and then preserved under − 80 °C. Each participant provided the written informed consent for participation. All cases enrolled in this work were naive to immunotherapy and radiotherapy before and after surgery. The present study was approved by the Medical Ethics Committee of Second Affiliated Hospital of Nanjing Medical University. The present study was performed following the 1975 Declaration of Helsinki.

**Table 1:** Association between clinicopathological features and Girdin expression in 57 patients with gastric cancer

Characteristics	Number of patients	Girdin low expression (< median)	Girdin high expression (≥ median)	*P* value
Number	57	27	30	
Ages (years)				0.550
< 45	28	13	15	
≥ 45	29	14	15	
Gender				0.561
Female	27	13	14	
Male	30	14	16	
Pathological stage				0.025
I-II	25	16	9	
III-IV	32	11	21	
Tumor size				0.013
< 3 cm	26	17	9	
≥ 3 cm	31	10	21	
Lymph node metastasis				0.353
Yes	30	13	17	
No	27	14	13	

### Cell culture and transfection

The GC cells (SGC-7901 and MGC-803) were provided by the Cell Bank of the Chinese Academy of Sciences (Shanghai, China), and cultured within RPMI-1640 (Gibco; Thermo Fisher Scientific, Inc., Waltham, MA, USA) that contained 10% fetal bovine serum (FBS; Invitrogen; Thermo Fisher Scientific, Inc.), followed by incubation under 5% CO_2_ and 37 °C. Besides, we acquired 3 Girdin-targeting siRNAs together with the negative controls from GenePharma Co., Ltd. (Shanghai, China). Later, the Lipofectamine® 2000 reagent (Invitrogen; Thermo Fisher Scientific, Inc.) was adopted for transfecting these plasmids into cells (5 × 10^6^) to 50 nM for 48 h. Moreover, Lipofectamine® 2000 was also used to transfect pCDNA3-Girdin into GC cells for 24 h to induce Girdin over-expression. Thereafter, we carried out quantitative real-time PCR (qRT-PCR) for validating Girdin expression.

### RT-qPCR

We used TRIzol reagent (Thermo Fisher Scientific, Inc.) for extracting total RNA from GC cells (1 × 10^7^) in line with specific protocols. We adopted TaqMan High-Capacity cDNA Reverse Transcription Kit, TaqMan Fast PCR Master Mix (Applied Biosystems; Thermo Fisher Scientific, Inc.), and related primers for qRT-PCR following specific protocols, with GAPDH being the endogenous reference for normalizing Girdin. The RT-qPCR procedure was conducted as follows: 5 min initial denaturation under 95 °C; then 10 s denaturation under 95 °C, 20 s annealing under 60 °C, and 10 s extension under 72 °C for 40 cycles. Thereafter, the 2^−ΔΔCq^ approach was applied in calculating relative gene expression based on previous description. Sequences of primers used in this study were shown below, for Girdin, 5'-GAGAAGCAGTGGTGGGTTCC-3' (forward) and 5'-GAGCAACACAGATGAACCGC-3' (reverse); for GAPDH 5'-TCCTCTGACTTCAACAGCGACAC-3' (forward) and 5'-CAC CCTGTTGCTGTAGCCAAATTC-3' (reverse).

### Cell proliferation detection

At 24 h after transfection, SGC-7901 and MGC-803 cells (5000/well) were inoculated into 96-well plates. After seeding for 24, 48, and 72 h, the Cell Counting Kit-8 (CCK8; Dojindo Molecular Technologies, Inc., Kumamoto, Japan) was employed to measure cell apoptosis. In addition, we utilized microplate spectrophotometer (Thermo Fisher Scientific, Inc.) for detecting absorbance (OD) value at 450 nm.

### Cell cycle analysis

At 48 h post-transfection, we collected SGC-7901 and MGC-803 cells, washed them by PBS, and then fixed them with ethanol under − 20 °C. After rinsing by PBS again, cells were subjected to rehydration and resuspension for 30 min into the 10 µl propidium iodide (PI)-RNase A solution (Sigma-Aldrich; Merck KGaA) under 37 °C. After staining, we used a flow cytometer (BD Biosciences, Franklin Lakes, NJ, USA) for measuring DNA content in 1 × 10^5^ cells. Finally, we utilized FlowJo 7.6.1 software (FlowJo LLC, Ashland, OR, USA) for result analysis.

### Cell apoptosis assay

We rinsed the harvested SGC-7901 and MGC-803 cells by cold PBS, followed by staining using the Annexin V-FITC apoptosis detection kits (Nanjing KeyGen Biotech Co., Ltd., Nanjing, China). Thereafter, we adopted a flow cytometer (BD Biosciences) to analyze apoptosis and employed FlowJo 7.6.1 software for result analysis.

### Transwell invasion analysis

We used Matrigel-coated (BD, Franklin Lakes, NJ, USA) Transwell chambers (pore size, 8-um; Costar, Manassas, VA, USA) to conduct invasion assay. At 48 h post-transfection, cells cultured in the FBS-free RPMI-1640 medium (200 μl) were added into Matrigel-coated (1 mg/ml, 30 μl) upper chamber coated with. Meanwhile, FBS-containing RPMI-1640 medium was added to lower chamber as the chemo-attractant. At 24 h post-incubation, cells locating onto membrane surface were scraped, then the scraped cells were rinsed by PBS, followed by 100% methanol fixation and Giemsa dye staining.

### Scratch assay

In scratch assay, SGC-7901 and MGC-803 cells (1 × 10^6^/well) were inoculated into the 6-well plates. When cells reached about 90% confluency, the pipette tip (1 ml) was utilized to make a wound on the cell monolayer surface. Then, images were obtained at 0 and 48 h under the microscope, respectively, to evaluate wound closure, which was determined by (scratch width at 0 h—scratch width at 24/48 h)/scratch width at 0 h × 100%. In addition, ImageJ software (NIH, USA) was used to measure the inter-edge distance.

### Western blotting analysis

We isolated the extracted total proteins through 10% SDS-PAGE, followed by transfer on PVDF membranes (EMD Millipore, Billerica, MA, USA). Thereafter, membranes were subjected to 5% skimmed milk powder blocking in Tris-buffered saline that contained 0.1% Tween-20 (TBS-T) under 37 °C for a period of 30 min, rinsed with TBS-T four times and then probed using primary antibodies under 4 °C overnight. In this assay, the primary antibodies (dilution, 1:1000) used were provided by Abcam (Cambridge, MA, USA), as shown below, anti-β-catenin, anti-GSK-3β, anti-Cyclin D1, anti-N-cadherin, anti-MMP-2, anti-β-actin, anti-LEF1. Later, the membranes were washed extensively and probed using HRP-labeled goat anti-rabbit IgG polyclonal secondary antibody (cat. no. 7074; dilution, 1:2000; CST Biological Reagents Co., Ltd.) under ambient temperature for 1 h. Later, enhanced chemiluminescence (Pierce; Thermo Fisher Scientific, Inc.) was conducted to measure the immunoreactivity, whereas the ChemiDoc XRS imaging system and analysis software (Bio-Rad Laboratories, Inc., Hercules, CA, USA) was utilized for visualization, with GAPDH being an endogenous reference.

### In vivo xenograft assays

Altogether twelve 6-week-old BALB/c nude mice were provided by Beijing HFK Bioscience Co. Ltd. (Beijing, China). Then, all animals were kept in the SPF room, as approved by the Committee of our hospital. To analyze tumor spread, each BALB/c nude mouse was given subcutaneous injection of MGC-803 cancer cells with NC transfection or shGirdin transfection (1 × 10^7^). Then, we determined tumor volume according to tumor volume = πab^2^/6 (where a indicates tumor length, whereas b indicate tumor width) at specific time points. After injection for 4 weeks, we determined the tumor weight. The Animal Care and Use Committees of Second Affiliated Hospital of Nanjing Medical University approved our study protocols, which were carried out according to related regulations and guidelines.

### Statistical analysis

Results were presented in a form of mean ± SD from 3 individual assays. IBM SPSS19.0 was employed for statistical analyses by ANOVA or Student’s *t*-test. Relationships of Girdin level in GC samples with clinicopathological characteristics were examined by Pearson’s chi-square tests. Also, we utilized the Kaplan–Meier method to analyze survival rate. A difference of *P* < 0.05 upon log-rank test was considered statistically significant.

## Results

### Girdin was over-expressed in GC samples

**Fig. 1:**
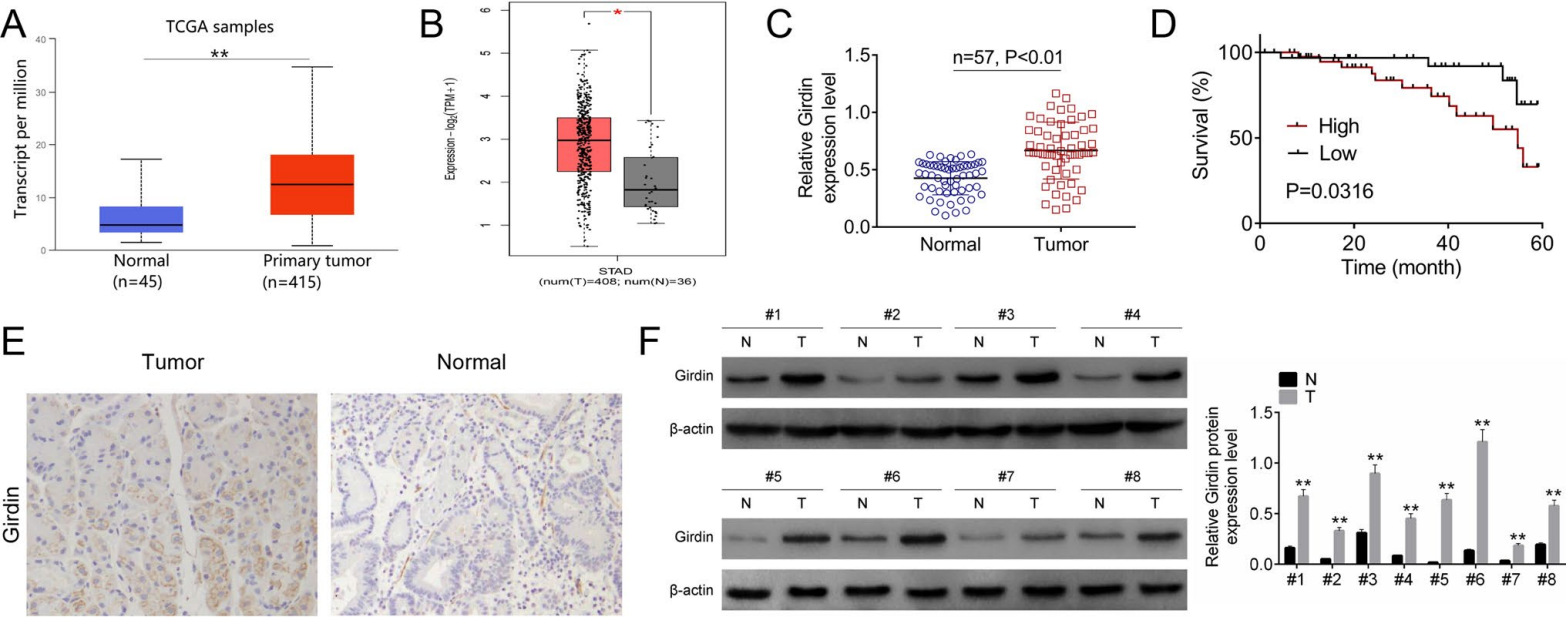
Girdin expression in GC clinical samples. (**A**) Girdin mRNA expression in GC tumor tissues and non-carcinoma samples in TCGA. (**B**) Girdin mRNA expression in GC tumor tissues and non-carcinoma samples in GEPIA. (**C**) Girdin mRNA levels in 57 GC tumor tissues and non-carcinoma samples were identified through qRT-PCR. (**D**) Girdin expression predicted an unfavorable survival rate. (**E**) The Girdin protein levels within GC and non-tumor samples were examined by IHC assay. (**F**) The expression of Girdin in eight paird GC samples was detected by western blot. Data are displayed in a form of mean ± SD. **P < 0.01 compared with N group

The expression of Girdin in TCGA and GEPIA database was explored, and as shown in Fig. 1A and B, Girdin was overexpressed in cancer tissues (*P* < 0.05). For exploring the effect of Girdin on GC, this study detected the levels of Girdin within GC samples and matched non-carcinoma samples through qRT-PCR and IHC assays. Figure 1C indicated that Girdin expression dramatically elevated within cancer tissues relative to matched non-carcinoma samples (*n* = 57, *P* < 0.01). According to the mean Girdin expression level, we classified all cases as high (*n* = 22) or low (*n* = 35) expression group, and determined their 5-year survival rates. It was shown that high Girdin expression predicted an unfavorable survival rate (*P* < 0.05, Fig. 1D). As revealed by IHC analysis, Girdin protein expression was upregulated within tumor tissues (Fig. 1E). The protein expression of Girdin in eight clinical sample of GC was examined by western blot. It was found that the protein expression of Girdin in GC tumor tissues was increased significantly compared with that in normal tissues (*P* < 0.05, Fig. 1F).

Moreover, this study analyzed those correlations between Girdin expression and clinicopathological parameters of GC patients. As a result, Girdin showed positive correlation with pathological stage and tumor size (*P* < 0.05, Table 1).

### Girdin knockdown suppressed cell growth and promoted cell apoptosis

**Fig. 2:**
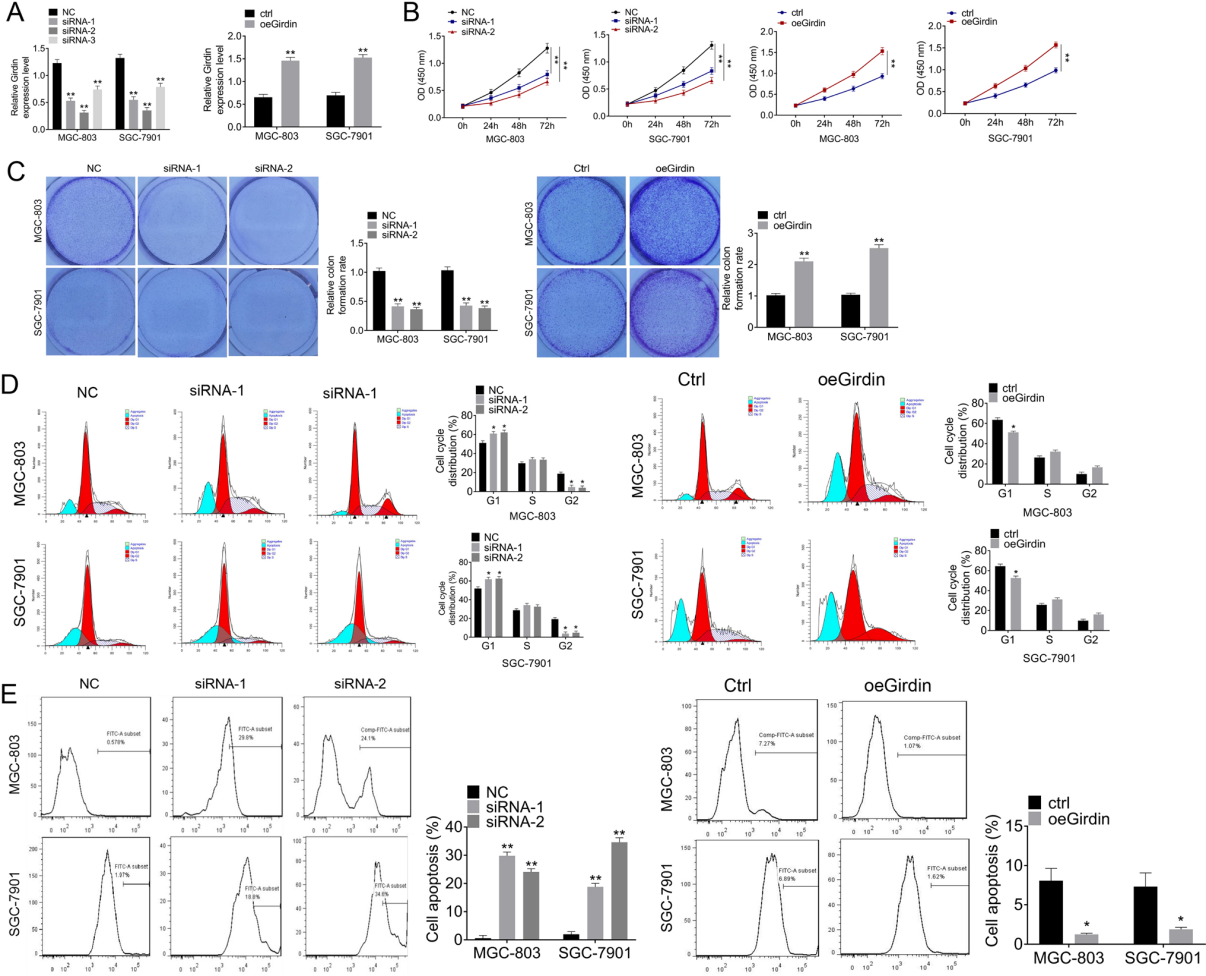
Knockdown of Girdin inhibits cell proliferation and promotes cell apoptosis. (**A**) SGC-7901 and MGC-803 cells were subjected to Girdin-targeting siRNAs or Girdin overexpression plasmids transfection, and the transfection efficiency was detected through qRT-PCR. (**B**) Cell proliferation was analyzed through CCK8 assay. (**C**) Cell colony formation was analyzed through crystal violet staining. (**D**, **E**) Cell cycle and cell apoptosis were analyzed through FCM. Data are presented in a form of mean ± SD. *P < 0.05

For examining the role of Girdin in GC cell characteristics, SGC-7901 and MGC-803 cells were transfected with Girdin-targeting siRNAs or Girdin overexpression plasmids, and qRT-PCR was conducted to detect transfection efficiency (Fig. 2A). It was shown that siRNA-1 and siRNA-2 had favorable effect on Girdin knockdown, which were used for further cell functional experiments. Thereafter, cell proliferation, cell colony formation, cell cycle, and apoptosis were examined. As shown in Fig. 2B and C, Girdin knockdown dramatically inhibited the cell viability and cell colony formation of SGC-7901 and MGC-803 cells (*P* < 0.05), while transfection with oeGirdin had opposite effects (*P* < 0.05). Moreover, downregulation of Girdin induced G1 stage arrest in the cell cycle of SGC-7901 and MGC-803 cells (*P* < 0.05), and oeGirdin transfection promoted the cell cycle progression (*P* < 0.05, Fig. 2D). Meanwhile, results of Annexin V-FITC assay indicated that siGirdin increased the apoptosis of MGC-803 and SGC-7901 cells (*P* < 0.05), and oeGirdin transfection had opposite effects (*P* < 0.05, Fig. 2E).

### Girdin silencing suppressed cell invasion and migration

**Fig. 3:**
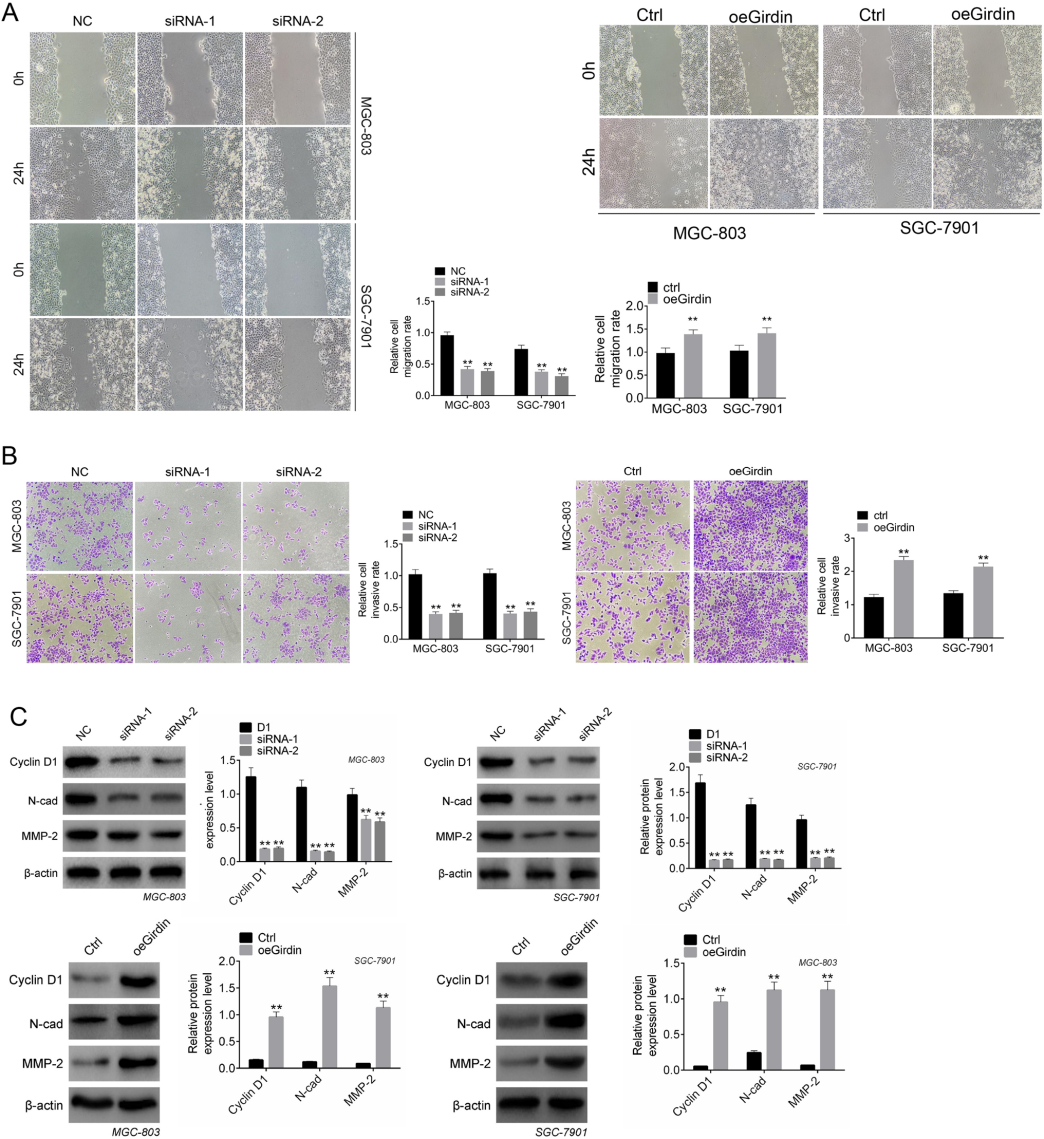
Knockdown of Girdin suppresses cell invasion and migration. (**A**) MGC-803 and SGC-7901 cell migration was evaluated through scratch assay. (**B**) MGC-803 and SGC-7901 cell invasion was assessed through Transwell assay. (**C**) Protein expression of Cyclin D1, N-cadherin, MMP-2 was examined by western blot. Data are displayed in a form of mean ± SD. **P < 0.01 compared with NC or Ctrl group

The present work also conducted scratch and Transwell assays to assess the Girdin effect on GC cell invasion and migration. As shown in Fig. 3A, Girdin silencing remarkably suppressed their migration (*P* < 0.01), and Girdin overexpression obviously promoted cell migration of SGC-7901 and MGC-803 cells (*P* < 0.01). Moreover, the results of Transwell assay revealed that Girdin knockdown significantly inhibited the invasion of SGC-7901 and MGC-803 cells relative to NC group (*P* < 0.05, Fig. 3B). Compared with Ctrl group, Girdin overexpression promoted the invasion of the above two cell lines (*P* < 0.05, Fig. 3B). N-cadherin is known as a biomarker in tumors that promotes cell migration and invasion. The expression of Cyclin D1, N-cadherin, MMP-2 was explored by western blot. As shown in Fig. 3B, siRNA of Girdin effectively inhibited the protein expression of Cyclin D1, N-cadherin, MMP-2 in GC cells, and Girdin overexpression promoted the expression of Cyclin D1, N-cadherin, MMP-2 in GC cells (*P* < 0.01, Fig. 3C).

### Girdin regulated AKT/GSK3β/β-catenin signaling

**Fig. 4:**
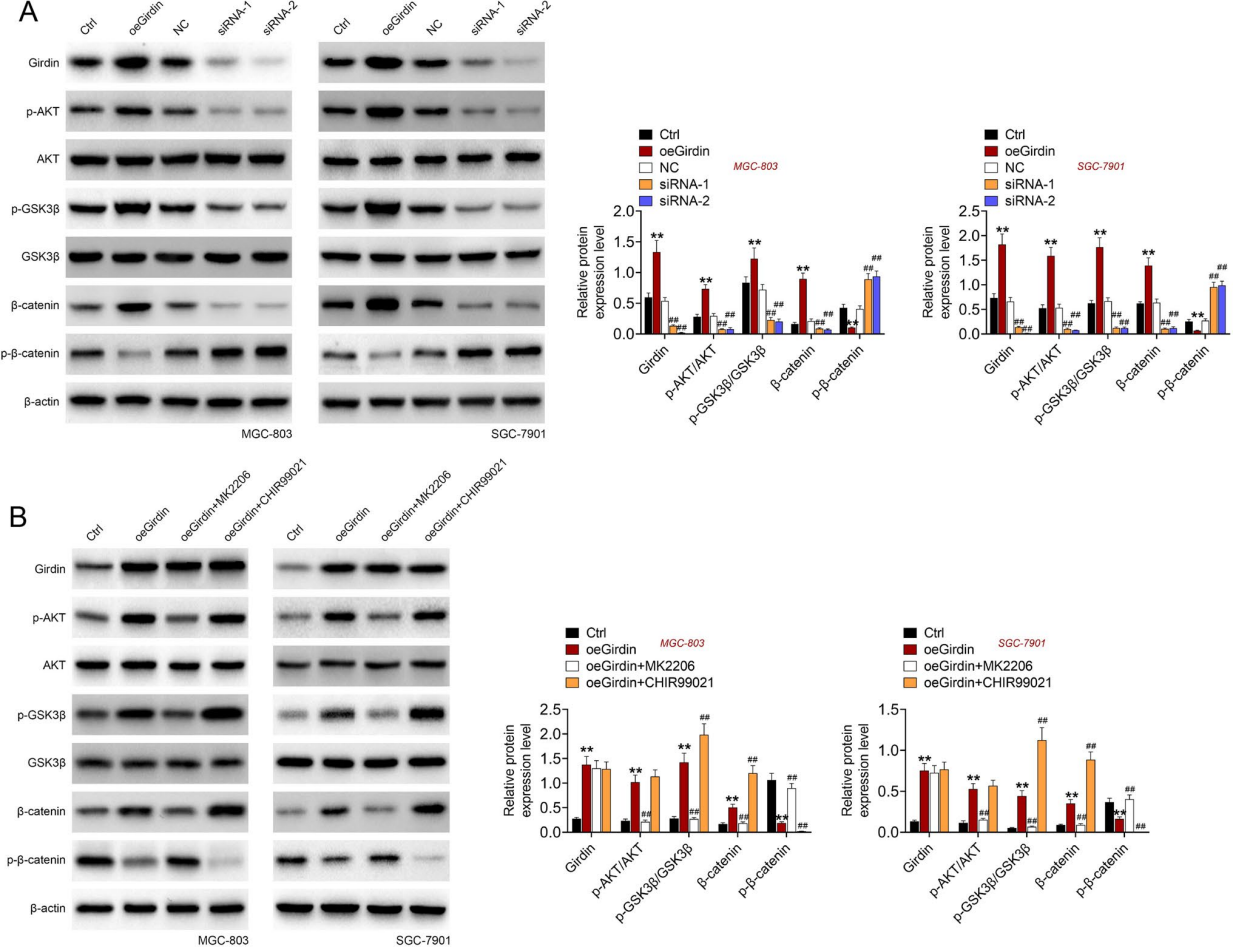
Girdin promotes cell phenotype of GC by regulating AKT/GSK3β/β-catenin signaling. (**A**) GC cells were transfected with Girdin overexpression or knockdown, and the expression of Girdin, p-AKT, p-GSK3β, AKT, GSK3β, β-catenin, and p-β-catenin was detected by western blot. (**B**) GC cells were transfected with Girdin overexpression and then with AKT inhibitor (MK2206) and GSK3β inhibitor (CHIR99021) treatment, the expression of Girdin, p-AKT, p-GSK3β, AKT, GSK3β, β-catenin, and p-β-catenin was detected by western blot. Data are displayed in a form of mean ± SD. **P < 0.01 compared with Ctrl, ^##^*P* < 0.01 compared with oeGirdin

To elaborate the mechanism of Girdin in promoting GC cell growth and migration, we performed western blot assay to detect the effect of Girdin on AKT/GSK3β/β-catenin signaling. As shown in Fig. 4A, Girdin overexpression stimulated the expression of p-AKT/AKT, p-GSK3β/GSK3β, and β-catenin, while inhibited the expression of p-β-catenin. Girdin knockdown showed the opposite effect on AKT/GSK3β/β-catenin signaling. AKT inhibitor (MK2206) and GSK3β inhibitor (CHIR99021) were subsequently employed to treat oeGirdin transfected GC cells. The expression of p-AKT/AKT, p-GSK3β/GSK3β, β-catenin, and p-β-catenin was tested by western blot. The results exhibited that MK2206 reversed the effect of oeGirdin, and CHIR99021 treatment enhanced the effect of oeGirdin on AKT/GSK3β/β-catenin signaling in MGC-803 and SGC-7901 cells (Fig. 4B).

### Girdin promoted the GC cell phenotype by regulating AKT/GSK3β/β-catenin signaling

**Fig. 5:**
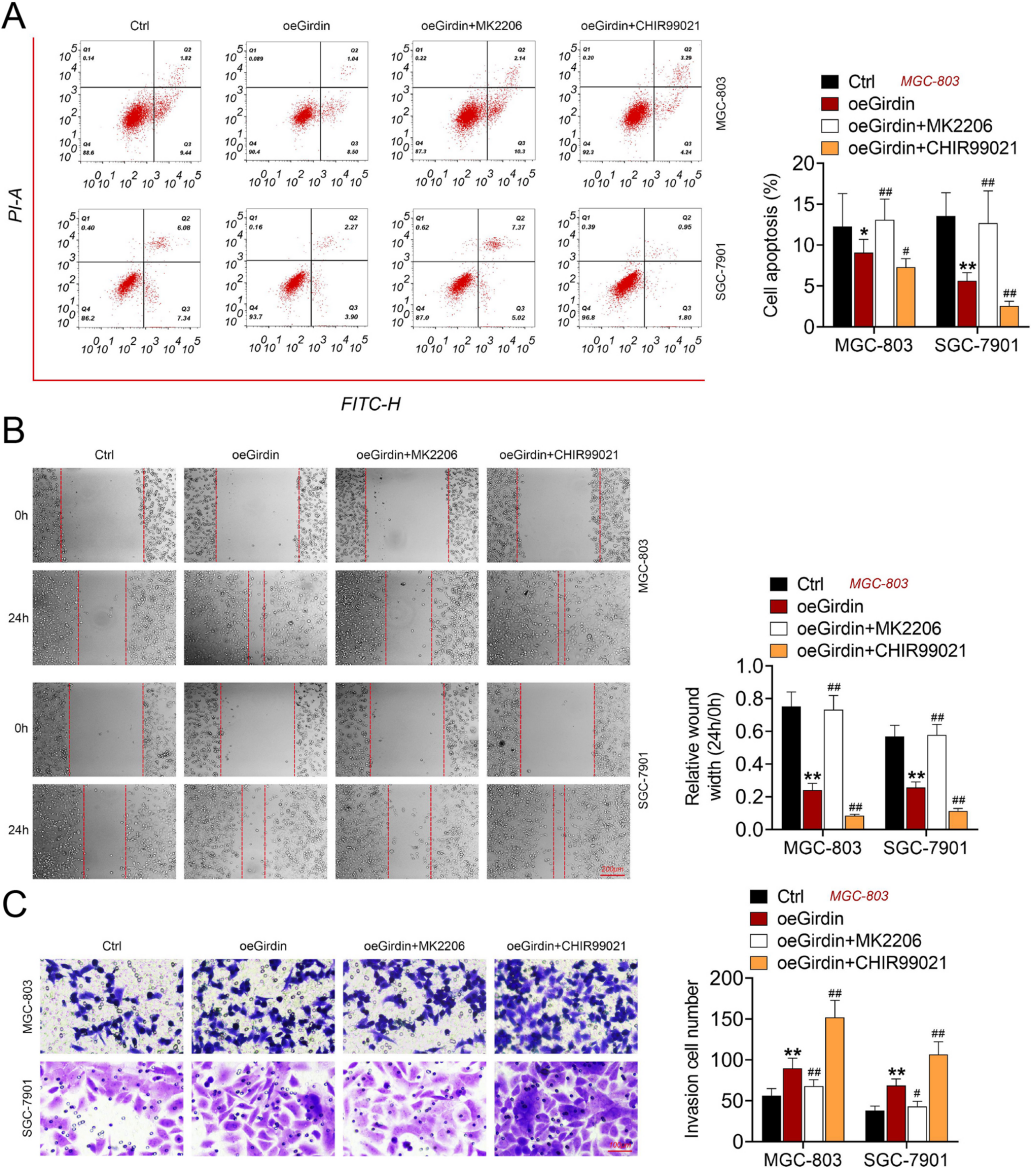
Girdin promoted the GC cell phenotype by regulating AKT/GSK3β/β-catenin signaling. GC cells were transfected with oeGirdin, GC cells were then treated AKT inhibitor (MK2206) and GSK3β inhibitor (CHIR99021). Cell apoptosis (**A**), migration (**B**) and invasion (**C**) were detected. Data are displayed in a form of mean ± SD. **P < 0.01 compared with Ctrl, ^##^*P* < 0.01 compared with oeGirdin

After transfected with oeGirdin, GC cells were then treated AKT inhibitor (MK2206) and GSK3β inhibitor (CHIR99021). Afterwards, the cell apoptosis, migration, and invasion were detected. The results showed that oeGirdin inhibited cell apoptosis and promoted cell migration and invasion (Fig. 5A–C). While, MK2206 treatment reversed the effect of oeGirdin, and CHIR99021 treatment enhanced the effect of oeGirdin on cell apoptosis, migration and invasion in MGC-803 and SGC-7901 cells (Fig. 5A–C).

### Girdin knockdown significantly restrained tumor growth in vivo

**Fig. 6:**
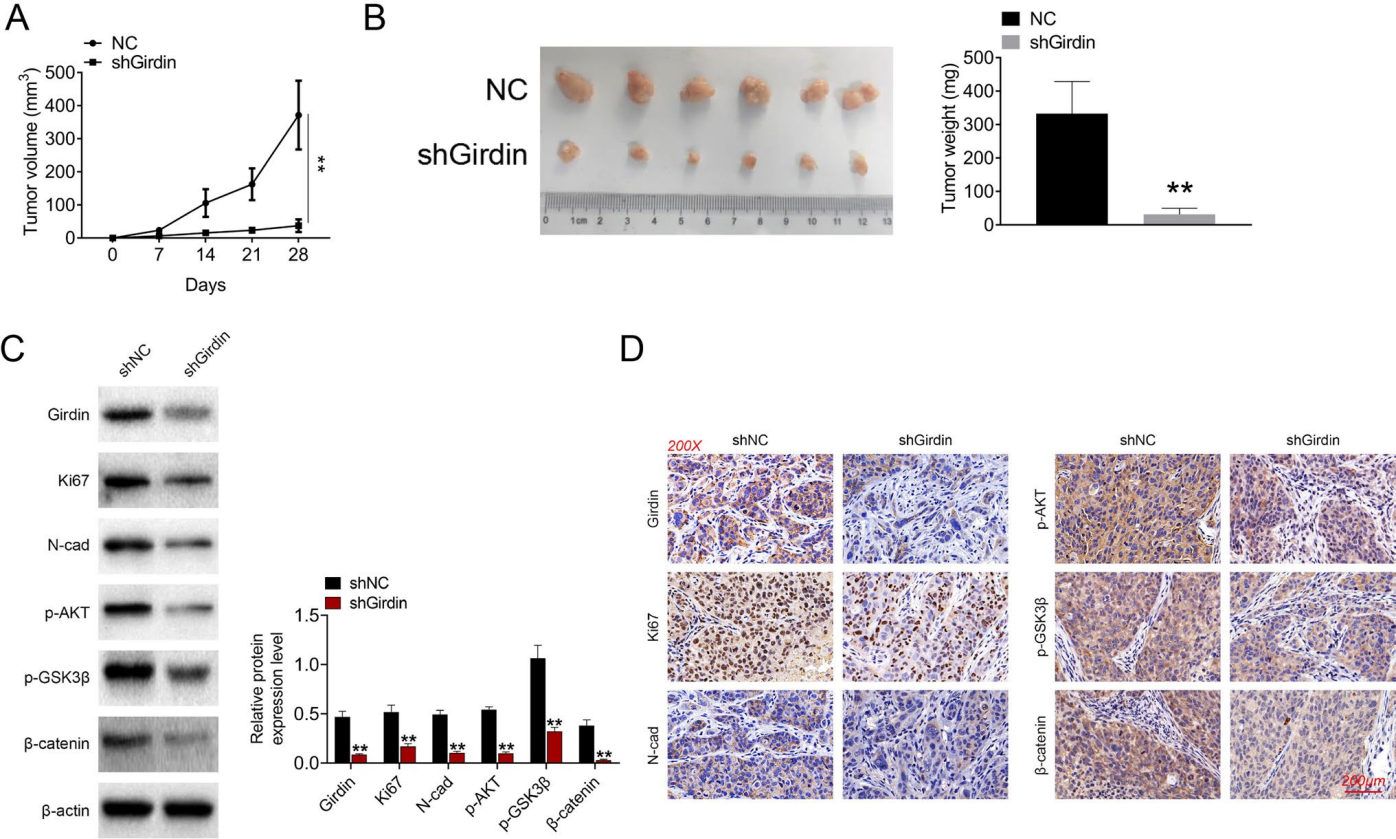
Girdin downregulation delays tumor growth in vivo. (**A**) Tumor volume was measured at intervals of 7 days for 35 days post-injection to construct the tumor growth curves. (**B**) Tumor weights separated from nude mice of every group were measured on day 35 post-injection. (**C**) Protein expression of Girdin, Ki67, N-cad, p-AKT, p-GSK3β, and β-catenin was explored by western blot. (**D**) Protein expression of Girdin, Ki67, N-cad, p-AKT, p-GSK3β, and β-catenin was explored by IHC. Data are exhibited in a form of mean ± SD. *P < 0.05 compared with NC group

At last, we examined the role of Girdin in tumor growth by a xenograft tumor model. In brief, nude mice were given subcutaneous injection of shGirdin- or shNC-transfected GC cells. Afterwards, we monitored tumor growth at intervals of 7 days. As a result, Girdin silencing remarkably postponed tumor growth in vivo (*P* < 0.01, Fig. 6A). After implantation for 5 weeks, we sacrificed all nude mice to collect the tumor tissues. Our results suggested that, Girdin silencing evidently reduced tumor volume and tumor weight (*P* < 0.01, Fig. 6B). The expression of Girdin, Ki67, N-cad, p-AKT, p-GSK3β, and β-catenin was explored by western blot and IHC, and the results indicated that shGirdin inhibited the expression of Ki67, N-cad, p-AKT, p-GSK3β, and β-catenin (*P* < 0.01, Fig. 6C, D).

## Discussion

Girdin is a momentous macromolecular protein in cells, which possesses a serine phosphorylation site at C-terminal 1416 (Takahashi et al. 2015). Girdin can specifically bind to the C-terminal of AKT, thus promoting the phosphorylation of AKT and kinase activity (Hayano et al. 2015). Besides, Girdin is found previously to to be related to neuroblastoma migration (Wang et al. 2011), actin remodeling (Enomoto et al. 2005), cell polarity (Ohara et al. 2012), angiogenesis (Kitamura et al. 2008), and autophagy. Recent studies have found that Girdin displays high expression level within cancers like lung cancer and GC, which predicts the dismal patient prognosis (Yang et al. 2018; Wang et al. 2018b). Choi et al. analyzed the protein expression of 892 GC cases by IHC, and found that Girdin was expressed in 289 cases (32.4%). Moreover, Girdin expression was remarkably related to tumor volume, advanced cancer, estrogen and progesterone receptor expression, and lymph node metastasis (LNM). For GC patients who abnormally expressed Girdin, the disease-free survival (DFS) and overall survival (OS) rates of which were remarkably decreased relative to those without Girdin expression. Therefore, the Girdin level is a potential factor to predict the prognosis of GC patients (Wang et al. 2014). Zhang et al. discussed the effect of Girdin silencing on colorectal cancer (CRC) cell sensitivity to oxaliplatin-based chemotherapy and the possible mechanism, and they discovered that Girdin silencing improved the sensitivity of GC cells to oxaliplatin by down-regulating TOP2B (Zhang et al. 2014). Ni et al. reported that Girdin was an actin binding protein, which exhibited high expression within glioma cells and was associated with cell migration (Ni et al. 2015). Moreover, they investigated that Girdin possibly modulated the PI3K/AKT signal transduction pathway to regulate glioma cell invasion and migration (Ni et al. 2015). We previously reported that Girdin showed high expression in pancreatic cancer patients, which was associated with their tumor stage and tumor size (Yang et al. 2020). Besides, Girdin silencing suppressed pancreatic cancer cell growth, invasion, and migration, while promoting their apoptosis (Yang et al. 2020). The present work examined the biological function of Girdin in GC cells. As a result, Girdin expression was increased significantly in GC tumor tissues, and the high Girdin expression predicted the unfavorable patient survival. Besides, Girdin upregulation showed positive correlation with pathological stage and tumor size. The results suggested the oncogenic role of Girdin in GC. Moreover, the result of IHC displayed that heterogenous Girdin expression was observed in tumor cells, which can be studied by single-cell transcriptomic analysis. Due to the limitations of experimental conditions and techniques, the heterogenous Girdin expression by single-cell transcriptomic analysis was not performed, which will be an important content of future research work.

Malignant tumor cells are characterized by unlimited proliferation, strong invasion, and migration (Zhao et al. 2013). This study also evaluated the effect of Girdin knockdown on GC cells. As a result, Girdin silencing apparently suppressed GC cell growth, migration and invasion, and enhanced cell apoptosis and cell cycle arrest. Besides, overexpression of Girdin had opposite effects on promoting GC cell growth, invasion and migration. These findings demonstrated the involvement of Girdin in enhancing GC progression. The expression of Cyclin D1, N-cadherin, MMP-2 in GC cells was also regulated by Girdin.

The underlying mechanism was subsequently explored, the results exhibited that Girdin promoted the phosphorylation of AKT, GSK3β, and β-catenin. Girdin could inhibit the phosphorylation of β-catenin. Previous study has indicated that activated GSK3β complex and β-catenin forms a complex, leading to the phosphorylation of β-catenin and then p-β-catenin is degraded (Dai et al. 2021). Whereas AKT mediated GSK3β Ser9 phosphorylation leads to the inhibition of GSK3β, and β-catenin is released (Liu et al. 2018). To prove this hypothesis, we subsequently used AKT inhibitor (MK2206) and GSK3β inhibitor (CHIR99021) to treat oeGirdin transfected GC cells. The results revealed that oeGirdin suppressed cell apoptosis and promoted cell migration and invasion. While, MK2206 treatment reversed the effect of oeGirdin, and CHIR99021 treatment enhanced the effect of oeGirdin on cell apoptosis, migration, and invasion in MGC-803 and SGC-7901 cells. These results proved that Girdin promoted the GC cell phenotype by regulating AKT/GSK3β/β-catenin signaling. Moreover, the in vivo experiments also illustrated that Girdin knockdown contributed to restraining tumor growth.

In conclusion, our study implicates that Girdin is abnormally expressed in GC samples, which irritates the development of GC by regulating the AKT/GSK3β/β-catenin signaling. These findings may shed novel lights on the molecular diagnosis and targeted therapy of GC.

## Data Availability

All data in the manuscript is available through the responsible corresponding author.
